# Genetic diversity within leukemia-associated immunophenotype-defined subclones in AML

**DOI:** 10.1007/s00277-021-04747-x

**Published:** 2022-01-13

**Authors:** F. Tiso, T. N. Koorenhof-Scheele, E. Huys, J. H. A. Martens, A. O. de Graaf, B. A. van der Reijden, S. M. C. Langemeijer, F. W. M. B. Preijers, L. I. Kroeze, J. H. Jansen

**Affiliations:** 1 Department of Laboratory Medicine, Laboratory of Hematology, Radboudumc, Nijmegen, The Netherlands; 2 Department of Molecular Biology, Radboud University, Nijmegen, The Netherlands; 3 Department of Hematology, Radboudumc, Nijmegen, The Netherlands; 4 Department of Pathology, Radboudumc, Nijmegen, The Netherlands

**Keywords:** AML, Immunophenotype, Molecular diagnostics, MRD

## Abstract

Acute myeloid leukemia (AML) is a highly heterogeneous disease showing dynamic clonal evolution patterns over time. Various subclones may be present simultaneously and subclones may show a different expansion pattern and respond differently to applied therapies. It is already clear that immunophenotyping and genetic analyses may yield overlapping, but also complementary information. Detailed information on the genetic make-up of immunophenotypically defined subclones is however scarce. We performed error-corrected sequencing for 27 myeloid leukemia driver genes in 86, FACS-sorted immunophenotypically characterized normal and aberrant subfractions in 10 AML patients. We identified three main scenarios. In the first group of patients, the two techniques were equally well characterizing the malignancy. In the second group, most of the isolated populations did not express aberrant immunophenotypes but still harbored several genetic aberrancies, indicating that the information obtained only by immunophenotyping would be incomplete. Vice versa, one patient was identified in which genetic mutations were found only in a small fraction of the immunophenotypically defined malignant populations, indicating that the genetic analysis gave an incomplete picture of the disease. We conclude that currently, characterization of leukemic cells in AML by molecular and immunophenotypic techniques is complementary, and infer that both techniques should be used in parallel in order to obtain the most complete view on the disease.

**Supplementary Information:**

The online version contains supplementary material available at 10.1007/s00277-021-04747-x.

## Introduction

Acute myeloid leukemia (AML) is a highly heterogeneous clonal disease. Heterogeneity is observed between and within patients morphologically, immunophenotypically, and genetically [1, 2] and different responses can be observed to the applied treatments. Immunophenotyping allows the detection of leukemic cell (sub)populations. When compared to healthy cells, malignant cells can be recognized by the presence of aberrant patterns of cluster of differentiation (CD) monoclonal antibodies (mAb), called leukemia-associated immunophenotypes (LAIPs) [3]. LAIPs can be used to follow the malignant cells in time, and enable the detection of measurable residual disease (MRD) after treatment. LAIPs are defined by the combination of markers directed against aberrant antigens on the AML cells. LAIPs defined by asynchronous expression of antigens or aberrant co-expression of cross-lineage antigens are often found. Less abundant and harder to follow up in time are LAIPs defined by the over- or under-expression of non-aberrant combinations of antigens and LAIPs defined by aberrant light scatter properties [4]. Multi-color flow cytometry currently allows the identification of patient-specific LAIPs in the vast majority of AML patients, and often several distinct LAIPs may be detected within an individual [3]. Next to LAIPs, different-from-normal (DfN) cell detection can also be used to identify the presence of MRD [5]. DfN cells are defined as cells which show aberrant markers or display an aberrant differentiation/maturation pattern that are new, compared to diagnosis. DfN cell detection can be used in MRD settings, especially when there is no immunophenotypic data from the moment of diagnosis or when there were no LAIPs detected at diagnosis. In addition to immunophenotyping, genetic analyses by cytogenetics and sequencing of leukemia-associated genes may reveal pathogenic genetic aberrations in the majority of AML cases, which are used for prognostic risk categorization and the choice of personalized, targeted therapies [6–8].

Although almost 80% of AML patients may reach a complete remission after intensive treatment, in about 50% a relapse develops [9, 10]. Monitoring the persistence of malignant cells during and after treatment allows the early recognition of an impending relapse and tailoring of post-remission therapy [5, 11]. MRD is currently investigated by flow cytometry, which can be applied at a sensitivity between 1:10^3^ and 10^4^ for most patients as well as by molecular methods aiming to detect the disease-associated mutations. Molecular analysis using next-generation panel sequencing can be broadly applied, but generally has limited sensitivity (1–5%). In contrast, Q-PCR-based assays are highly sensitive allowing the detection of residual malignant cells with a sensitivity of 1:10^5^–10^6^. A drawback of the latter is that specific assays need to be optimized for each individual mutation. Currently, sensitive assays for nucleophosmin 1 (*NPM1*) and *PML-RARA, RUNX1-RUNX1T1* and *CBFB-MYH11* fusions are broadly used [10]. Previous studies revealed that genetically different subclones populating the bone marrow of myelodysplastic syndrome (MDS) and AML patients may co-exist at the same time. In addition, these subclones may show different evolutionary trajectories in time and respond differently to therapy [12]. Even though immunophenotyping and next-generation sequencing are widely used, their complementarity and overlap are still under investigation. The aim of our study was to compare how the two techniques characterize the malignancy, at the moment of diagnosis. In order to do that, we genetically investigate the different LAIPs which could be isolated from each patient, using the diagnostic panels.

## Materials and methods

### Patient samples

Bone marrow (BM) samples from AML patients were collected at diagnosis. Mononuclear cells were isolated using density gradient centrifugation (Ficoll) and cryopreserved. All selected patients had signed informed consent. The study was conducted in accordance with the Declaration of Helsinki and institutional guidelines and regulations from the Radboudumc Nijmegen (IRB number: CMO 2013/064). The patient characteristics are listed in Supplementary Table 1.

### Sorting of AML subclones

MNCs from BM were thawed and stained as reported in the supplementary material and methods. The monoclonal antibodies (Moabs) were selected based on the LAIPs found at diagnosis. For each patient, specific gating-strategies were used for identification of their LAIP populations. The specific gating-strategy as well as the used Moabs was first tested on a Navios Flow Cytometer (Beckman Coulter), and afterwards, the different subpopulations were sorted on a BD FACSAria II SORP cell sorter and represented in Figs. 2, 3 and 4. Only populations for which at least 5000 cells were sorted were further analyzed.

**Fig. 1:**
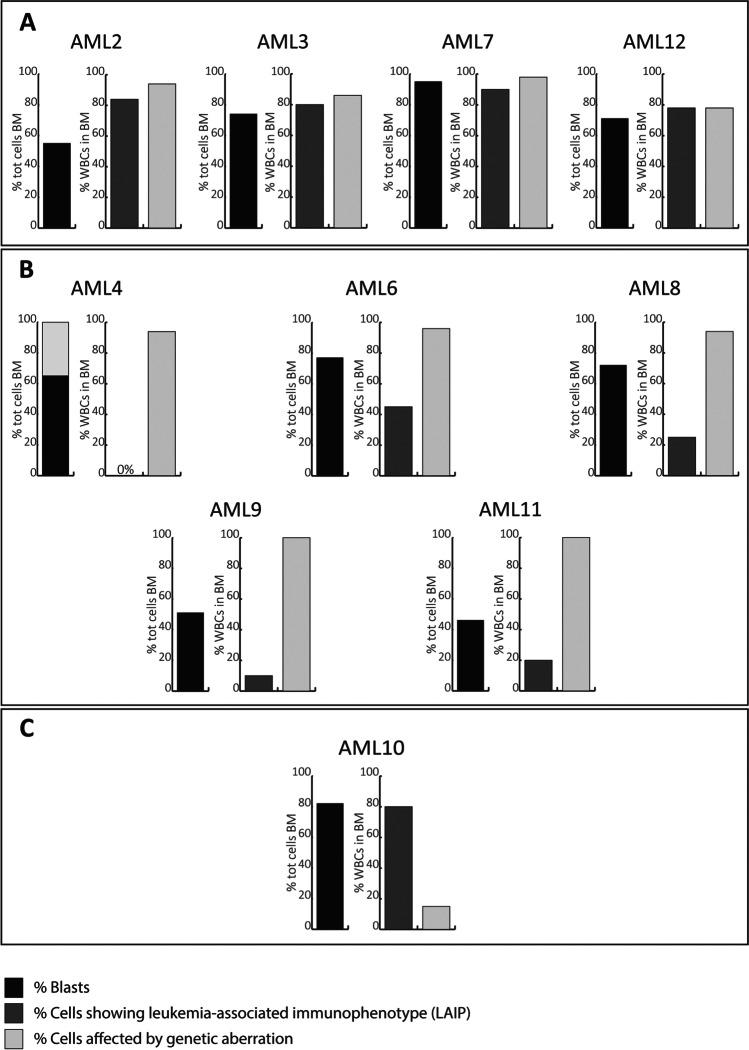
Each bar plot indicates the percentage of cells which were identified as blasts (from the morphological analysis), within the full bone marrow smear (tot cells BM); LAIPs (from the IFT analysis) and carrying genetic aberrations (from the molecular analyses), within the white blood cells (WBC). In **A**, **B**, and **C**, the patients belonging to the three identified scenarios are represented. **A** Percentage of WBCs carrying genetic aberration is comparable to the percentage of WBCs detected to be LAIPs. **B** Percentage of WBCs carrying genetic aberration is higher than the percentage of WBCs detected to be LAIPs. **C** Percentage of WBCs by genetic aberrations is lower than the percentage of WBCs detected to be LAIPs

### DNA isolation and amplification

DNA was isolated using NucleoSpin Blood QuickPure kit (Macherey Nagel, Düren, Germany) or NucleoSpin Tissue XS (Macherey Nagel, Düren, Germany), according to the manufacturer’s protocol. When the extraction yield was insufficient (< 5 µg), 5 µl of DNA was amplified using the Qiagen REPLI-g kit (Qiagen, Venlo, The Netherlands) according to the manufacturer’s protocol. All amplifications were performed in duplicate. The detailed protocol can be found in the supplementary materials and methods.

### DNA sequencing

The full protocol’s descriptions can be found in the supplementary materials and methods. All the fractions were sequenced using a panel of single-molecule tagged molecular inversion probes (smMIPs) covering target regions in 27 myeloid and lymphoid malignancy-associated driver genes (Table S3). Libraries were prepared as previously described (Supplementary material [13]), and the sequencing was performed on the Illumina NovaSeq 6000 or NextSeq500 platform (Illumina, San Diego, CA). Each sample was sequenced in duplicate to exclude artefacts caused by the amplification procedure. The indicated variant allele frequencies (VAFs) are the mean VAFs of the duplicates. The variability in VAFs between duplicates was calculated in 316 samples, and it was estimated to be ± 1.4%. CEBPA and PTPN11 (exons 3 and 13) were sequenced using the PacBio sequencing technique. Libraries were prepared according to the PacBio® Barcoded Adapters for Multiplex SMRT® Sequencing protocol (PacBio), and the samples were run on PacBio Sequel performing a single molecule real-time (SMRT) sequencing. The variability in VAF between duplicates was calculated in 40 samples (comparing the VAFs detected for each SNP in the different samples) and resulted to be ± 6.4%. To detect internal tandem duplication in fms like tyrosine kinase 3 (*FLT3*-ITD) gene, we used fragment length analysis by capillary electrophoresis. Wild type product size was 328 bp. *FLT3*-ITD relative mutant level was calculated using the area under the peak. The VAF was calculated dividing the area under the peak of the mutated signal to the sum of the area under the peak of the wild type and mutant signals.

## Results

**Fig. 2:**
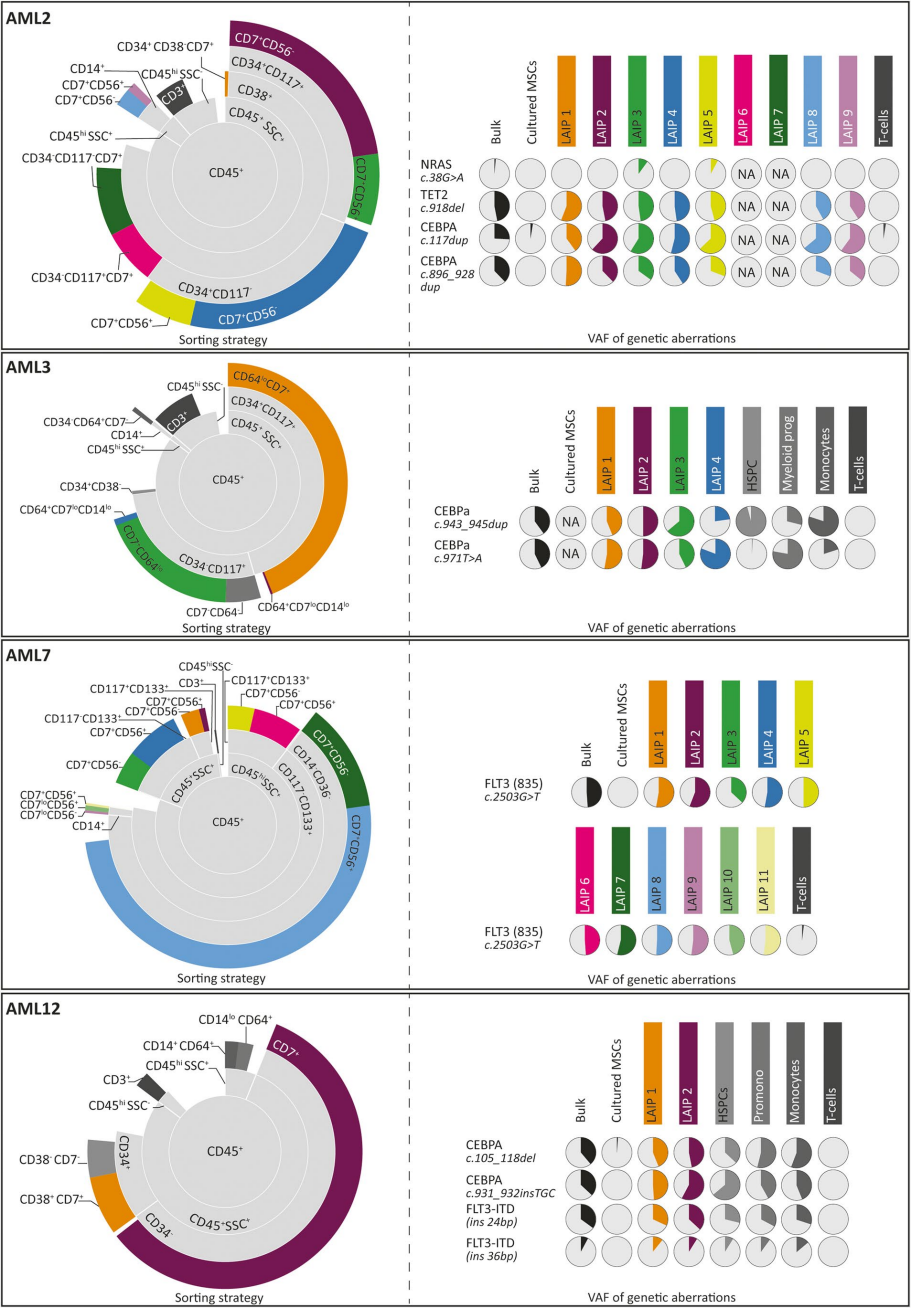
Sorting strategy and genetic characterization of AML patients in which the percentage of WBCs carrying genetic aberration is comparable to the percentage of WBCs detected to be LAIPs. On the left side of each panel, the sorting strategies applied for the 10 patients is indicated. Each circle represents the fraction of cells showing a certain marker (indicated within the circle). A complete circle represents 100% of the cells. The light-grey circles represent the hierarchical gating strategy used to identify the populations of interest. We first selected the WBC based on CD45 + , which is depicted as 100% of the cells. The outermost circles indicate the sorted populations. The colored circles indicate the sorted LAIPs, whereas the circles in the different shades of grey indicate the immunophenotypically non-aberrant sorted populations. The same populations are represented with the same colors on the right side of each figure, and the populations’ names are specified. For each patient, T cells were sorted and used as non-tumor control, together with primary cultured MSCs. LAIPs were numbered from the most immature to the most mature, when interpretable. In the right side of the panel, the results of the molecular analyses are represented. The rows indicate the different mutations and the columns indicate all the sorted subfractions. The VAFs of the detected mutations are represented as pie charts in which the colored part of the pies indicates the VAF of each specific mutation in each different subpopulation. When fusion genes are tested with Q-PCR, only + or − is indicated because we could not quantify the VAF. Whenever a sample could not be tested, non applicable is indicated by NA

**Fig. 3:**
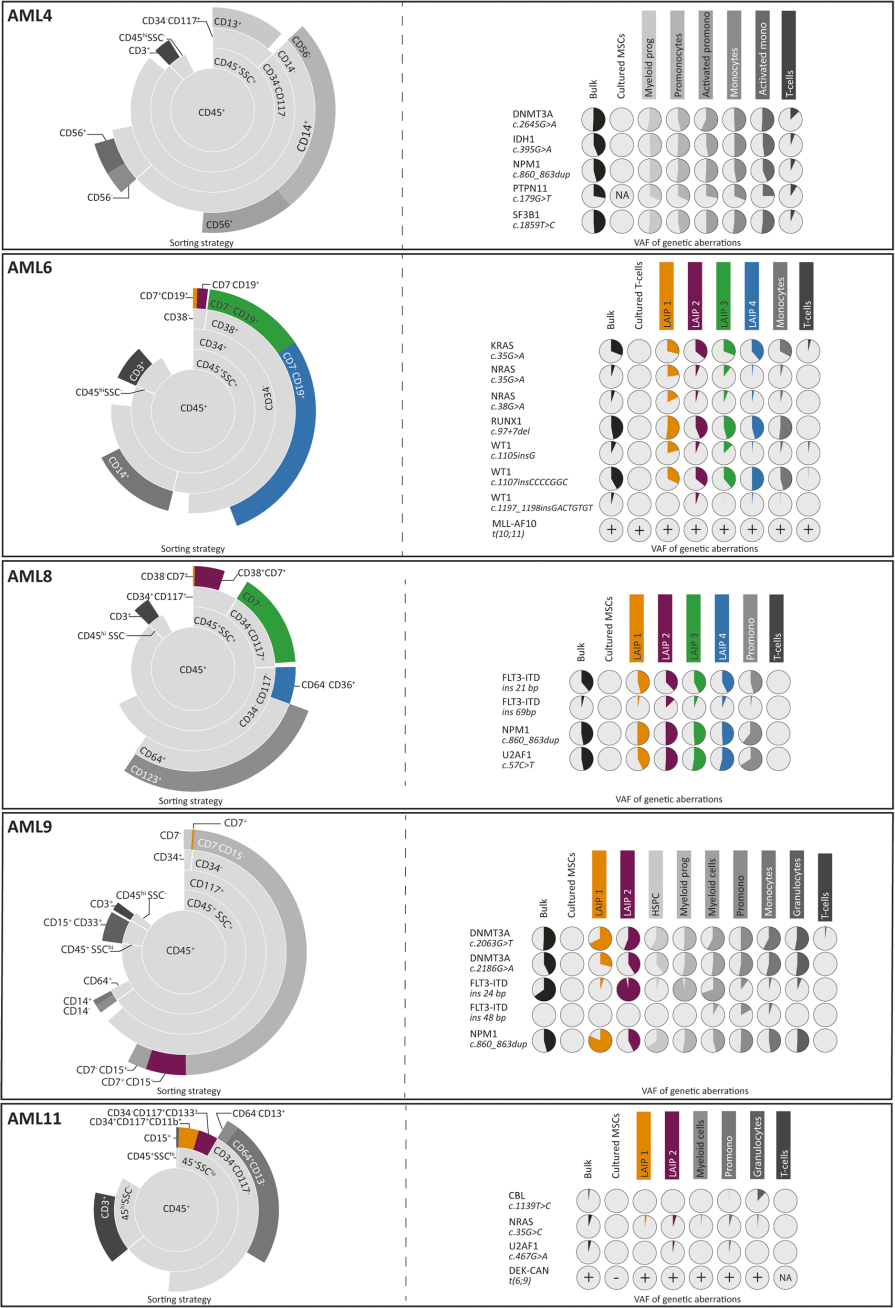
Sorting strategy and genetic characterization of AML patients in which the percentage of WBCs carrying genetic aberration is higher than the percentage of WBCs detected to be LAIPs. The structure of the figure is the same as reported in the description of Fig. 2

**Fig. 4:**
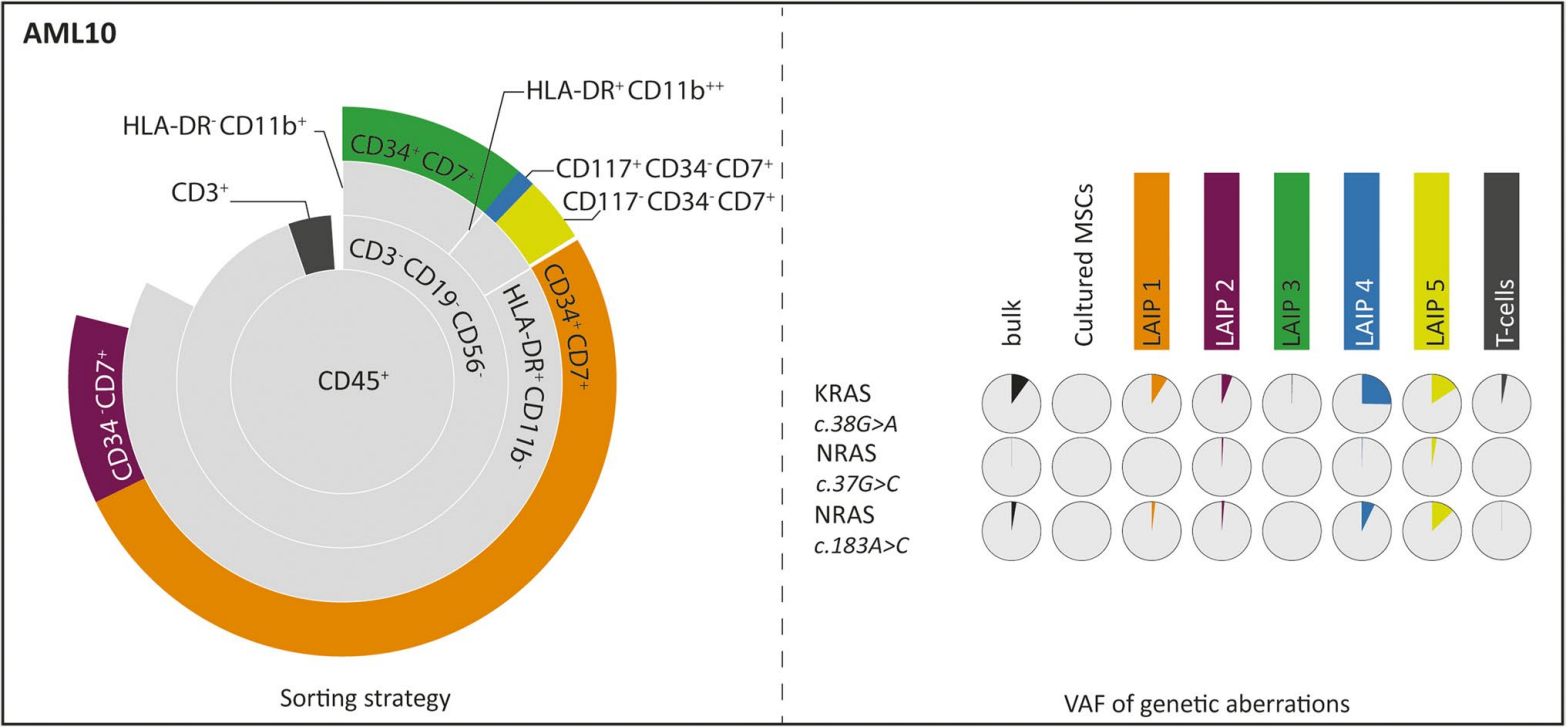
Sorting strategy and genetic characterization of AML patients in which the percentage of WBCs carrying genetic aberrations is lower than the percentage of WBCs detected to be LAIPs. The structure of the figure is the same as reported in the description of Fig. 2

First, we compared the differences in characterizing the malignancy in 10 different AML cases, depending on the applied technique. The percentage of blast cells defined by morphological analysis ranged from 40 to 95%, whereas the percentage of LAIPs detectable ranged from 0 to 90% (Fig. 1). The number of distinct LAIPs found in individual patients ranged from 0 to 7 (Figs. 2, 3, and 4). For each patient, a personalized Moab panel was designed allowing the sorting of all individual LAIPs, as well as immunophenotypically normal populations (Table S2). For the sorting experiments, we used frozen bone marrow samples. As expected, granulocytes were less represented in the thawed samples, but no large differences were found between LAIPs that were present in the samples analyzed at the moment of diagnosis, before Ficoll and freezing was performed, compared to the spectrum and size of LAIP populations analyzed in the corresponding frozen samples, indicating that no preferential loss of particular LAIPs occurred during freeze-thawing (Figs. 1, 2, 3, and 4). For each patient, a different set of somatic mutations was found, with an average of 3.7 mutations per patient (Figure S1). Mutational load was expressed as variant allele frequency (VAF), representing the percentage of aberrant sequence reads at the locus being analyzed. The gene with the highest VAF was considered to represent the major leukemic clone which was represented in Fig. 1. All mutations were autosomal, and no loss of heterozygosity was seen in the affected loci based on SNP analysis (not shown) and karyotyping (Table S1). Therefore, all mutations were assumed to be heterozygous and the percentage of cells carrying the mutation was calculated as twice the VAF. To study the similarities and differences between leukemia subclones defined by immunophenotyping and mutational status, we performed an in-depth analysis of the bone marrow of 10 AML patients (Table S1). LAIPs were determined for all cases using the Moab panels for the diagnostics of AML. In three of the cases, almost the entire myeloid compartment was immunophenotypically aberrant. Consequently, apart from the different LAIP-populations, only immunophenotypically normal T cells were isolated. In the other seven patients, the myeloid compartment also contained various immunophenotypically normal populations which were sorted as well. For the 10 patients, in total, 86 fractions were collected and sequenced using a next-generation panel for mutations (Figs. 2, 3, and 4).

We performed error-corrected targeted deep sequencing for 27 leukemia-associated driver genes using single-molecule molecular inversion probe (smMIP) technology (Table S3). In addition, *CEBPA* and *PTPN11* mutations were assessed using PacBio sequencing and *FLT3-ITD* mutations were analyzed using quantitative fragment length analysis by capillary electrophoresis. In case of chromosomal translocations reported by cytogenetics, we also investigated the presence of gene fusions in the different subfractions using Q-PCRs. Cultured mesenchymal stem cells (MSCs) and sorted T cells were analyzed as non-tumor controls. Mutations in T cells were identified at low VAFs for some of the mutations (Figs. 2, 3, and 4). This might represent contamination occurring during the sorting procedure but, in some cases, the presence of mutated T cells was confirmed by sequencing cultured T cells (Supplementary table 4), indicating that part of the T cells were derived from leukemic progenitor cells.

When comparing the percentage of cells determined to be LAIPs by immunophenotyping to the percentage of cells that carried mutations, we could distinguish three scenarios, based on the data collected at diagnosis (Fig. 1). The first scenario (Figs. 1A and 2) is represented by four cases (AML2, AML3, AML7, and AML12). For these, the percentage of cells showing an aberrant immunophenotype approximately equaled the percentage of cells that carried one or more mutations. All the different LAIPs and immunophenotypically normal fractions from these cases were sorted and genotyped (Fig. 2). In 2 of the 4 patients (AML2 and AML3), multiple genetic clones were observed, showing that different LAIPs may have a different genetic make-up. The opposite was noticed in the other two cases (AML7 and AML12), where different LAIPs belonged to the same genetic clone. In the patients belonging to this category, the disease is equally well characterized by both techniques and could therefore be followed over time using either method, provided that sensitive assays for several of the mutations are available. Multiple mutations were only present in a small subclone of the entire leukemia population (*NRAS* mutation in AML2, 36 bp ins *FLT3-ITD* in AML12), since in both cases those subclones are derived from bigger subclones, the use of the other markers to keep track of the disease is more efficient for adequate MRD detection.

The second scenario (Figs. 1B and 3) is represented by five cases (AML4, AML6, AML8, AML9, and AML11) in whom the percentage of cells carrying a mutation was substantially higher than the percentage with an aberrant immunophenotype. In one case, (AML4) no LAIP was identified, whereas more than 95% of the cells carried mutations. All the immunophenotypically non-aberrant populations were found, also in these cases, to carry genetic aberrancies. Also, within this category, heterogeneity could be observed. In AML4 and AML8, two genetically defined subclones could be observed, distinguished by the acquisition of a mutation in PTPN11 in AML4 and by a 68-bp insertion in FLT3 in AML8. In the rest of the patients, instead, multiple genetic subclones could be identified. For the cases in this group, molecular analyses would allow a more complete surveillance of MRD, as they cover a larger part of the leukemic population, at the moment of diagnosis. For each of the patient, one of the identified mutations which is present in all the cells could be used as marker in order to follow the disease in time. The third scenario (Figs. 1C and 4) is represented by one patient (AML10). While immunophenotyping detected aberrations in approximately 80% of the bone marrow cells of this patient, mutations were only seen in 20% of the cells. In this scenario, the malignancy is much better characterized by immunophenotyping than by the applied molecular technique, indicating that the panel of mutations that was analyzed was incomplete.

## Discussion

In nine of the ten AML cases, at least one genetic mutation was present at a VAF of approximately 50% (and thus 100% of the cells) within the isolated LAIP clones, indicating a good correlation between the aberrant genotype and phenotype. In one case (AML10), however, the sorted LAIPs contained a mutation only in a fraction of the aberrant cells, indicating that in that case, the mutational screening likely failed to identify a major pathogenic mutation. This may be improved by broadening the screening panel of mutations analyzed at diagnosis and by increasing the depth of the detection limit of NGS, which at the moment is 1–5% for most of the commonly applied sequencing methods. In addition, our data showed that in seven cases, mutations were also clearly present in populations that were non-aberrant using the standard immunophenotyping panels. This implies that characterization of the malignancy at the moment of diagnosis by standard immunophenotyping may be incomplete, and that, potentially, relapses may be missed. Further expansion of the panel of monoclonal antibodies that are used may at least partially solve this issue [14]. Some mutations correlate with the presence of certain CD markers. One known correlation is NPM1 mutations that correlate with CD34- blasts [15]. The NMP1 mutated cases in our cohort (AML4, AML8, and AML9) were all myelo-monocytic leukemias (that indeed were CD34 negative). Currently, detection of MRD is commonly performed making use of the LAIPs that are defined at diagnosis. This can be broadly applied, as in most cases of AML, indeed, one or more LAIP populations can be defined. The inclusion of DfN cells in the assessment of MRD can increase the number of patients in whom are suitable for MRD detection through IFT can be performed. MRD detection by genetic analysis is not yet commonly applied for all mutations. For many other molecular targets, standardized assays that allow the detection of malignant cells at the level of 1/10^4^ to 1/10^5^ are still lacking. Early therapeutic intervention based on the detection of MRD after treatment was clearly shown to be beneficial in several studies [16]. Our data show that with the currently used standard immunophenotypic and molecular analysis, disease characterization at diagnosis is incomplete. Although we did not measure this in MRD samples, this creates the possibility that relapses might be missed, even when both techniques are used simultaneously. Both techniques can further be ameliorated by the inclusion of more immunophenotypic markers and further expansion of gene panels. Furthermore, more precise data need to be acquired on the prognostic value of MRD. This is highlighted by the recent observation that in some cases, a complete clinical remission after induction and consolidation therapy was observed, while still very large (premalignant) clones were present harboring TET2 or DNMT3A mutations [11]. In addition, very low numbers of AML cells bearing an *t*(8;21) [17] or inv(16) [18] (detected by Q-PCR for the corresponding fusion transcripts) may be present for many years without leading to relapse of the disease. This is in contrast to acute promyelocytic leukemia, in which the presence of very low amounts of PML-RAR positive cells after therapy almost invariably leads to relapse of the disease [19]. Therefore, the relevance of the presence of low numbers of (pre)malignant cells in relation to the development of relapse may be dependent on the mutations that are present and needs to be further defined. We conclude that currently, detection of the malignancy at the moment of diagnosis by molecular and immunophenotypic techniques is complementary; we recommend that both should be used in parallel, also for MRD detection during follow-up, in order to obtain the most complete view on resistant disease and early detection of relapse. At the same time, both techniques should be further developed to enhance the prognostic value and justification of clinical interventions.

## Supplementary Information

Below is the link to the electronic supplementary material.

Supplementary file1 (DOCX 281 KB)
