# Supplementary material for: Genetic diversity within leukemia-associated immunophenotype-defined subclones in AML
Source: Ann Hematol. 2022 Jan 13;101(3):571–9. doi: 10.1007/s00277-021-04747-x (PMC8810467; doi:10.1007/s00277-021-04747-x)
Supplement: Supplementary file 1 — Supplementary file1 (DOCX 281 KB) [file 277_2021_4747_MOESM1_ESM.docx]

**Genetic diversity within leukemia-associated immunophenotype-defined subclones in AML**

Tiso F.¹, Koorenhof-Scheele T. N.¹, Huys E.¹, Martens J. H. A.^2^, de Graaf A. O.¹, van der Reijden B. A.¹, Langemeijer S. M. C.^3^, Preijers F. W. M. B.¹, Kroeze L. I.^4^, Jansen J. H.¹

¹Department of Laboratory Medicine, Laboratory of Hematology, Radboudumc, Nijmegen, The Netherlands, ^2^Department of Molecular Biology, Radboud University, Nijmegen, The Netherlands, ^3^Department of Hematology, Radboudumc, Nijmegen, The Netherlands, ^4^Department of Pathology, Radboudumc , Nijmegen, The Netherlands.

**Correspondence:**

Prof. Dr. J. H. Jansen

Laboratory of Hematology, Department of Laboratory Medicine, Radboud University Medical Center, Radboud Institute for Molecular Life Sciences

PO Box 9101, 6500 HB Nijmegen, The Netherlands;

e-mail: Joop.Jansen@Radboudumc.nl

**Acknowledgements:** This work was supported by a grant from the Dutch Cancer Society (grant

#10813). The authors have no conflicting interests with respect to the content of this paper.

**SUPPLEMENTARY DATA**

**Supplementary Materials and Methods**

**Patient samples.** Bone marrow (BM) samples from AML patients were collected at diagnosis. Mononuclear cells were isolated using density gradient centrifugation (Ficoll) and cryopreserved. All selected patients had signed the informed consent. The study was conducted in accordance with the Declaration of Helsinki and institutional guidelines and regulations from the Radboudumc Nijmegen (IRB number: CMO 2013/064). The patient characteristics are listed in Table S1.

**Sorting of AML subclones.** MNCs from BM were thawed in the presence of 100 ul DNAse I (2 mg/µM) and incubated for 10 min in a solution of 1.6 ml fetal calf serum (FCS), 10 ul heparin (5,000U/ml) and 100 ul MgSO4 (0.22µM). Subsequently, the cells were washed with PBS with 1% FCS and 2mM EDTA centrifuging for 5 minutes at 600 g. Subsequently cells were stained with different combinations (Table S2) of MoAb-conjugates (CD3 FITC, CD3 PE-Cy7, CD7 FITC, CD7 PE, CD11b APC, CD13 ECD, CD13 PE, CD14 PB, CD14 ECD, CD15 PB, CD19 PE Cy7, CD33 A750, CD33 APC, CD34 A750, CD34 A700, CD36 APC-A700, CD38 APC, CD38 A750, CD38 ECD, CD38 Cy5.5, CD56 PECy7, CD64 ECD, CD64 PE-Cy7, CD117 PE-Cy7, CD117 PC5.5, CD117 APC, HLA-DR ECD from Beckman Coulter; CD13 BV421, CD15 FITC, CD16 PE, CD56 PE, CD135 PE, CD117 PE, CD123 PE from BD and 7AAD from Sigma Aldrich). The MoAbs were selected based on the LAIPs found at diagnosis. The in-house diagnostic practice for AML determination consists of a screening with an AML panel (CD2, CD3, CD4, CD5, CD7, CD8, CD10, CD11b, CD13, CD14, CD15, CD16, CD19, CD20, CD22, CD33, CD34, CD36, CD38, CD41, CD42, CD45, CD56, CD61, CD64, CD71, CD117, CD123, CD133, CD135, CD138, CD235a, cytTdT, cyt-MPO, NG2, IgKappa, Iglambda, HLA-DR). Based on the presence of aberrant markers within one patient’s myelo-blast population, one or more LAIPs can be established. Only markers expressed for >10% on the total leukemia blast population are used to call a LAIP. LAIPs are then confirmed with a specific staining, investigating only the markers detected to be aberrant within the myeloblast population. For each patient specific gating-strategies were used for identification of their LAIP populations. The specific gating-strategy as well as the used monoclonal antibodies (Moab’s) was first tested on a Navios Flow Cytometer (Beckman Coulter) and afterwards the different subpopulations were sorted on a BD FACSAria II SORP cell sorter and represented in Figure 1. Only populations for which at least 5000 cells were sorted, were further analysed.

**Mesenchymal stromal cell culture**. MSC lines were generated from nine out of ten patients. MSCs were used as germline control. BM-MNCs were seeded at a density of 10 to 23 x 10^4^ cells cm^-2^ in α-MEM medium (Sigma-Aldrich, St Louis, MO, USA) supplemented with heparin (3.5 IU/ml) and 5% platelet lysate. Platelet lysate was obtained by freeze-thawing of platelets (40 x 10^9^ platelets per ml) which were subsequently centrifuged at 4700 g after which the supernatant was collected. After 7 days of culture, the medium was refreshed. Cells were passed when they reached 80% confluency. MSCs were cultured up to 5 passages.

**T cell culture.** For one patient (AML6) MSC culture failed, so T cells were cultured and used as control. T cells were obtained by in vitro expansion of PBMCs. First monocyte depletion was performed by adherence to tissue culture 6-well plate. The non-adherent cells were cultured and expanded for 10 days in IMDM medium (Life Technology) with 10% human serum (PAA Laboratories GmbH, Pasching, Austria), interleukin-2 (100 IU/ml) and CD3/CD28-coated Dynabeads (Thermo Fisher). The purity of the T cells was measured by flow cytometric analysis using the CD3 surface marker, which reached 95%. To increase the purity of the population, the cells were also sorted, staining them with the same marker and reaching a final purity of 98%.

**DNA isolation and amplification**. DNA was isolated from the sorted subfractions and as well from the bulk, MSCs and T cells. DNA isolation was performed in two different ways, depending on the number of cells. If the total cell number was 30,000 or more, DNA isolation was performed using NucleoSpin Blood QuickPure kit (Macherey Nagel, Düren, Germany). In case the number of cells was less than 30.000, DNA isolation was performed using NucleoSpin Tissue XS (Macherey Nagel, Düren, Germany), in both cases according to the manufacturer’s protocol. When the extraction yield was insufficient (<5 µg) as measured with the Qubit fluorometer Quant-iT dsDNA HS Assay Kit (Thermo Fisher Scientific, Waltham, MA, USA), 5 µl of DNA was amplified using the Qiagen REPLI-g kit (Qiagen, Venlo, The Netherlands) according to the manufacturer’s protocol. All amplifications were performed in duplicate.

**Targeted deep sequencing using a myeloid gene panel.** All the (sorted) fractions were sequenced using a panel of single molecule tagged molecular inversion probes (smMIPs) covering target regions in 27 myeloid and lymphoid malignancy-associated driver genes (Table S3). Samples were sonicated with a Covaris E210 sonicator (Covaris, Inc. MA, USA). Libraries were prepared as previously described (Supplementary material^1^) and the sequencing was performed on the Illumina NovaSeq 6000 or NextSeq500 platform (Illumina, San Diego, CA). Each sample was sequenced in duplicate to exclude artefacts caused by the amplification procedure. Error-correction, alignment to the GRCh37 (hg19) genome and variant calling were performed using SeqNext module of the Sequence Pilot version 4.4.0 (JSI medical systems, Ettenheim, Germany). The following settings were used for variant calling using Sequence Pilot: Required Coverage/Min abs. cov., 20 combined, Mutations/Min abs. cov., 5 combined, Min % cov., 1% per dir. For most of the genes that were sequenced the minimal variant allele frequency which was called was ≥5%. For ASXL1, CALR and NPM1, instead, the minimal allele frequency called was 1%. Once a mutation was found in a subfraction of a patient, the sequences of all the subfractions were manually checked at the specific locations where the mutations were identified, in order to detect the mutations in the subfractions at VAFs lower than 5% or 1%, respectively. In order to discriminate a true variant from artifacts caused by the amplification procedure, a mutation needed to be found in both amplification duplicates. Moreover, for amplified samples we discarded duplicate variants with a VAF ≤5%, as it is not possible to discriminate between a true mutation or an artifactual duplication of a nucleotide at lower VAF levels due to the amplification procedure. We also discarded variant calls that were found in < 10 reads. The indicated VAFs are the mean VAFs of the duplicates. The variability in VAFs between duplicates was calculated in 316 samples and it was estimated to be ±1.4%.

**PacBio targeted deep sequencing**. CEBPA and PTPN11 (exon 3 and 13) were sequenced using the PacBio sequencing technique. CEBPA and PTPN11 show recurrent mutations in exon 3 and 13 in AML. PCR was used to amplify the relevant regions in both genes. The PCR reactions were performed using a long range PCR protocol and AmpliTaq Gold 360 mastermix (ThermoFisher), with different sets of primers (CEBPα FW- TGTAAAACGACGGCCAGTGGGCGAGCAGGGTCTCC, RV- CAGGAAACAGCTATGACCCCCAGGGCGGTCCCACAGC, for exon 3 of PTPN11 FW-GACCTTTGTGTTGAGTTGGTTGAC, RV - TAGGCATCCAAAGCCATCCAAG and for exon 13 of PTPN11 FW- TGACATCATCAGAGAGAAAGGTGG, RV- TAGCTTCAGATGGAAAGGCTC). The fragments were amplified for 10 minutes at 95°C, 35 cycles of 30 seconds at 94°C, 30 seconds at 60°C and 1 minute at 72°C and a final extension of 10 minutes at 72°C. Libraries were prepared according to the PacBio® Barcoded Adapters for Multiplex SMRT® Sequencing protocol (PacBio) and the samples were run on PacBio Sequel performing a Single Molecule Real-Time (SMRT) sequencing. The sequences were afterwards mapped to the GRCh37 (hg19) genome and the variants were called using SeqNext module of the Sequence Pilot version 4.4.0 (JSI medical systems, Ettenheim, Germany). The used Ensemble references were for CEBPA: [ENST00000498907](http://grch37.ensembl.org/Homo_sapiens/Transcript/Summary?db=core;g=ENSG00000245848;r=19:33790840-33793470;t=ENST00000498907) and for PTPN11: [ENST00000351677](http://grch37.ensembl.org/Homo_sapiens/Transcript/Summary?db=core;g=ENSG00000179295;r=12:112856718-112947717;t=ENST00000351677). The variability in VAF between duplicates was calculated in 40 samples (comparing the VAFs detected for each SNPs in the different samples) and resulted to be ±6.4%.

**Fragment length analysis by capillary electrophoresis.** This technique was used to determine the presence of internal tandem duplication on exon 14 (NM_004119) of fms like tyrosine kinase 3 (*FLT3*-ITD) gene. 50 ng of genomic DNA was amplified in a total volume of 25 μL containing 1× manufacturer's buffer, 1 mM MgCl_2_, 200 μM dNTPs, 10 pmol each of primers (FW 5’-GTAAAACGACGGCCAGCCGCCAGGAACGTGCTT-3’ (FAM), RV 5’-CAGGAAACAGCTATGACGATATCAGCCTCACATTGCCCC-3’) and 0.02 unit of AmpliTaq® DNA Polymerase (Applied Biosystems®). The PCR was performed starting with 11 minutes at 94°C followed by 37 cycles of 30 seconds at 94°C, 1 minute at 57°C and 2 minutes at 72°C, with a final extension of 10 minutes at 72°C. The PCR products were subsequently analysed on an ABI 3730 capillary electrophoresis machine (Applied Biosystems). Wild type product size was 328 bp. FLT3-ITDs relative mutant level was calculated using the area under the peak. The VAF was calculated dividing the area under the peak of the mutated signal to the sum of the area under the peak of the wild type and mutant signals.

**RNA isolation.** In two patients a chromosomal rearrangement was reported at diagnosis, which gives rise to a fusion transcript suitable for sensitive detection by PCR. From these cases, RNA was isolated from all the subfractions. RNA was isolated with NucleoSpin® RNA Plus (Machery-Nagel) when the number of cells was higher than 1x10^6^, otherwise Quick-RNA Miniprep (Zymo Research) was used. RNA extraction was performed according to the manufacturers’ protocols.

**Quantitative Reverse Transcription Polymerase Chain Reaction (qRT-PCR).** qRT-PCR was performed to detect DEK-CAN (patient AML11) and MLL-AF10 (patient AML6) fusion genes. cDNA was produced in triplicate using Moloney Murine Leukemia Virus Reverse Transcriptase (M-MLV RT) (Invitrogen). qRT-PCRs were performed using TaqMan™ Gene Expression Master Mix (Applied Biosystem™) and predesigned TaqMan Gene Expression Assays primer/probes, respectively DEK-CAN (assay number, #4331182) and MLL-MLLT10 (assay number, #4331182) (Thermo Fisher), using an Applied Biosystems 7500 real-time PCR machine. Measurements were performed in duplicate. A fusion gene was assumed to be present when measured with a Ct<40.

**SUPPLEMENTARY TABLES**

**Table S1.** Patients features, Cytogenetics (NA in case of non-applicable), morphological subtype, WBC count and % blasts in bone marrow.

**Table S2.** Different panels of MoAbs used to sort LAIPs from the 10 individual patients

**Table S3.** Genes included in smMIPs panel, including the targeted regions

**Table S4.** VAF of the **g**enetic mutations identified in the 10 AML patients in cultured T cells VS sorted T cells

**SUPPLEMENTARY FIGURE**

**
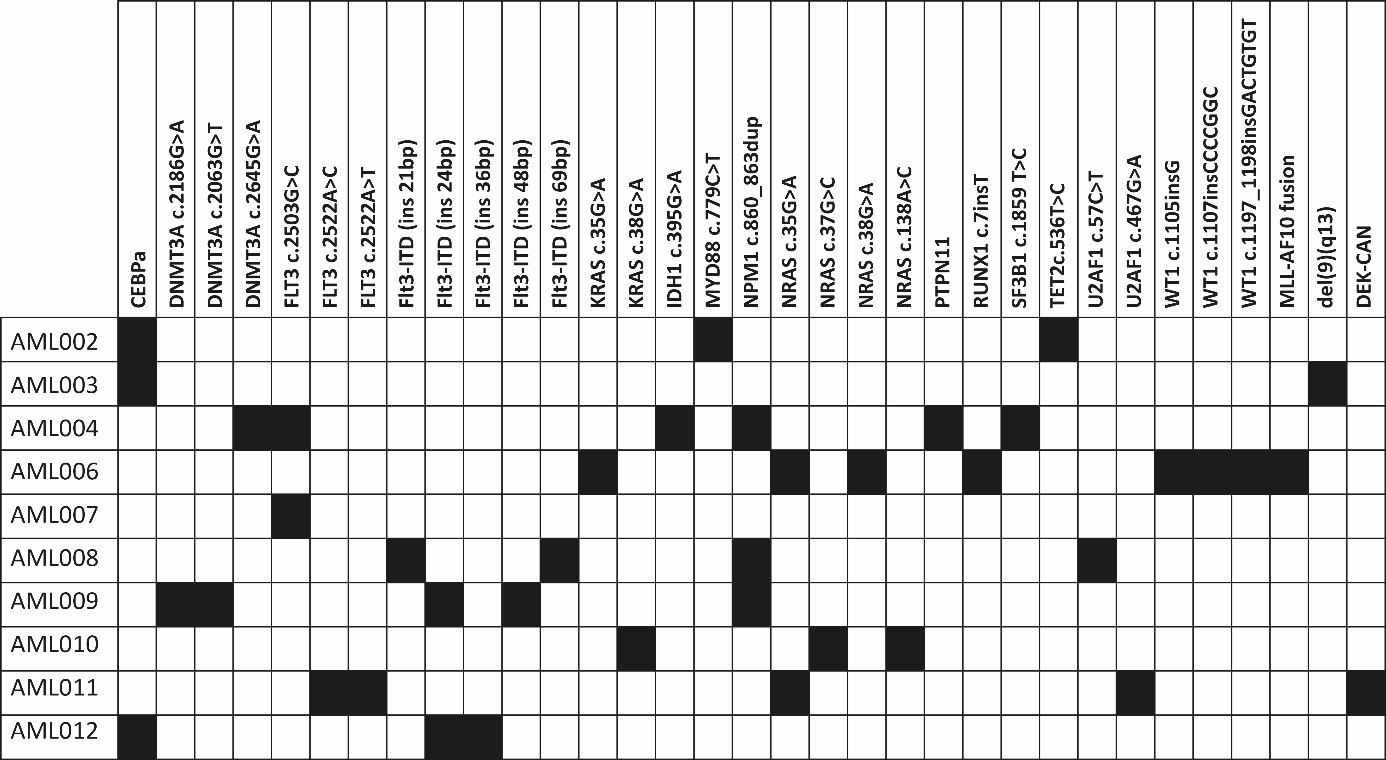
**

**Figure S1 -** **Detected genetic mutations.** Schematic representation of the different genes in which mutations and chromosomal aberrancies were found (black squares) in the various patients. Information on the karyotype and cytogenetic abnormalities found at diagnosis are shown in the last four columns. NA indicates non-applicable.

**Reference**

1 van Zeventer, I. A. *et al.* Mutational spectrum and dynamics of clonal hematopoiesis in anemia of older individuals. *Blood* **135**, 1161-1170, doi:10.1182/blood.2019004362 (2020).
